# Clade composition of a plant community indicates its phylogenetic diversity

**DOI:** 10.1002/ece3.6170

**Published:** 2020-03-13

**Authors:** Martin Bitomský, Pavla Mládková, Robin J. Pakeman, Martin Duchoslav

**Affiliations:** ^1^ Department of Ecology and Environmental Sciences Palacky University Olomouc Czech Republic; ^2^ Department of Ecology Czech University of Life Sciences Prague Czech Republic; ^3^ The James Hutton Institute Aberdeen UK; ^4^ Department of Botany Palacky University Olomouc Czech Republic

**Keywords:** biodiversity, clade index, phylogenetic divergence, phylogenetic regularity, phylogenetic richness

## Abstract

Phylogenetic diversity quantification is based on indices computed from phylogenetic distances among species, which are derived from phylogenetic trees. This approach requires phylogenetic expertise and available molecular data, or a fully sampled synthesis‐based phylogeny. Here, we propose and evaluate a simpler alternative approach based on taxonomic coding. We developed metrics, the clade indices, based on information about clade proportions in communities and species richness of a community or a clade, which do not require phylogenies. Using vegetation records from herbaceous plots from Central Europe and simulated vegetation plots based on a megaphylogeny of vascular plants, we examined fit accuracy of our proposed indices for all dimensions of phylogenetic diversity (richness, divergence, and regularity). For real vegetation data, the clade indices fitted phylogeny‐based metrics very accurately (explanatory power was usually higher than 80% for phylogenetic richness, almost always higher than 90% for phylogenetic divergence, and often higher than 70% for phylogenetic regularity). For phylogenetic regularity, fit accuracy was habitat and species richness dependent. For phylogenetic richness and divergence, the clade indices performed consistently. In simulated datasets, fit accuracy of all clade indices increased with increasing species richness, suggesting better precision in species‐rich habitats and at larger spatial scales. Fit accuracy for phylogenetic divergence and regularity was unreliable at large phylogenetic scales, suggesting inadvisability of our method in habitats including many distantly related lineages. The clade indices are promising alternative measures for all projects with a phylogenetic framework, which can trade‐off a little precision for a significant speed‐up and simplification, such as macroecological analyses or where phylogenetic data is incomplete.

## Graphical abstract

We demonstrate that clade composition of a community is a good indicator of phylogenetic diversity. We provide three indices that do not require phylogenies to estimate phylogenetic diversity (phylogenetic richness, divergence, and regularity).

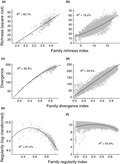

## 1 INTRODUCTION

The concept of phylogenetic diversity has revolutionized research in nature conservation and community ecology, as it enables the setting of conservation priorities or helps to identify which community assembly processes may have structured a community (Faith, 1992; Webb, Ackerly, McPeek, & Donoghue, 2002). Phylogenetic diversity estimation is based on phylogenetic distances (the amount of time since the most common ancestor of a pair of species), which are derived from dated phylogenies. Researchers have developed more than 70 metrics for quantifying alpha (within‐site) and beta (among sites) phylogenetic diversity, which are summarized under several frameworks (Scheiner, Kosman, Presley, & Willig, 2017; Tucker et al., 2017). It is worth noting that there is no agreement on the best or the most suitable metric. Phylogenetic diversity reflects diversification of lineages, geographic movement of lineages, and deep‐past and present assembly processes (Gerhold, Carlucci, Proches, & Prinzing, 2018; Webb et al., 2002; Yguel et al., 2016) that can be lineage specific (Elliott, Waterway, & Davies, 2016; Ndiribe et al., 2013). Considering such complexity, it is not possible to address phylogenetic patterns in communities using only one number. Therefore, this plethora of metrics is inevitable because each metric was designed to capture a specific aspect of phylogenetic diversity. Fortunately, various phylogenetic diversity metrics tend to correlate (Swenson, 2014; Vellend, Cornwell, Magnuson‐Ford, & Mooers, 2011) suggesting redundancy of some of them, thus, there has been an attempt to select a leading measure for each dimension of phylogenetic diversity (richness, divergence, and regularity; sensu Tucker et al., 2017; Table 1).

**Table 1:** Summary of three dimensions of phylogenetic diversity (defined by Tucker et al., 2017)

Dimension	Richness	Divergence	Regularity
Leading metric	Faith's phylogenetic diversity (Faith's PD)	Mean pairwise distance (MPD)	Variation of pairwise distances (VPD)
Mathematical function	Sum	Mean distance	Variation
Indicator of	Total evolutionary history	Similarity (phylogenetic relatedness)	Distribution of phylogenetic similarity
Main use	Conservation, predictor of future evolutionary potential	Proxy of trait similarity, test of habitat filtering versus limiting similarity	Testing competitive interactions
Example of a community with high value	Species‐rich communities	Clade‐rich communities	Communities with low asymmetric competition

To construct dated phylogenies requires considerable effort, and the whole process is affected by methodological biases and subjective decisions (Jantzen et al., 2019; Li et al., 2019). Further, calculated phylogenetic diversity metrics depend on the attributes of phylogenies, such as the degree of balance, diversification rate, resolution, taxon sampling, or tree reconstruction methods (Jantzen et al., 2019; Park, Worthington, & Xi, 2018; Swenson, 2009; Vellend et al., 2011). Here, we propose and evaluate an approach based on the idea of considering species phylogeny as a categorical variable (i.e., affiliation to a phylogenetic clade) rather than continuous (i.e., phylogenetic distances among species). A similar approach based on taxonomic relatedness (derived from a hierarchical Linnaean classification with applied taxonomic weights proportional to the level of the taxonomic rank two species hold in common, i.e., genus, family, or order) has proven to be useful to estimate biodiversity patterns in fish communities (Campbell, Neat, Burns, & Kunzlik, 2010; Hall & Greenstreet, 1998; Warwick & Clarke, 1995). There is also a clear parallel in functional ecology, clades can be considered as analogous to plant functional types (PFT) and their proportions can be utilized to indicate phylogenetic diversity of a community. Such a categorical approach to phylogeny might be a tool for ecologists who are not specialists in phylogenetics and might be useful in communities where some taxa do not have available DNA sequences or in studies where a little precision can be traded‐off for significant speed‐up and simplification.

This framework certainly causes a loss of information as we basically introduce a polytomy at a node of a defined clade, i.e. the categorical approach still separates species according to their clade affiliation, but it ignores phylogenetic information within clades. On the other hand, there is some indirect support that this loss of phylogenetic information within clades would have a marginal effect. Li et al. (2019) compared purpose‐built phylogenies (estimated from sequence data) with published synthesis‐based supertrees (which usually have more polytomies than the former) and showed that phylogenetic diversity metrics computed from both types of phylogenies were highly correlated. Cadotte (2015) also demonstrated that changing branch lengths did not strongly affect relationships between phylogenetic diversity and ecosystem function, suggesting that phylogenetic diversity measures are not so sensitive to the branch lengths of the phylogeny as long as the topology is right. One important criterion for choosing among metrics is their conceptual and mathematical simplicity (Vellend et al., 2011). Therefore, if the categorical approach provides sufficiently correlated values with other phylogeny‐based measures, than its use can be justified in order to simplify and speed‐up phylogenetic diversity estimation.

**Figure 1:**
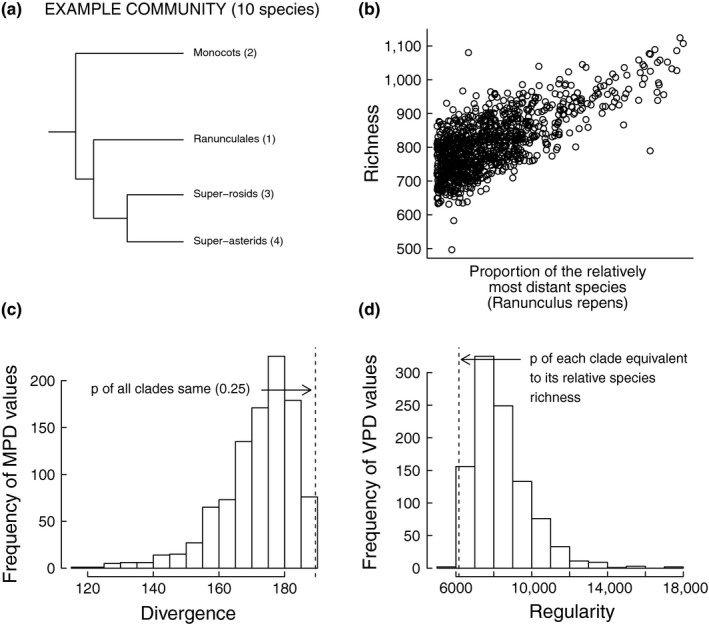
A conceptual example demonstrating how clade proportions (relative cover) affect values of leading metrics of all dimensions of phylogenetic diversity (Faith's PD = richness, MPD = divergence, and VPD = regularity). (a) We randomly selected 10 species: two monocots (*Agrostis capillaris* L. and *Bromus erectus* Huds.), one Ranunculales (*Ranunculus repens* L.), three superrosids (*Fragaria viridis* Weston, *Trifolium pratense* L., and *Vicia cracca* L.), and four superasterids (*Aegopodium podagraria* L., *Centaurea jacea* L., *Campanula patula* L., and *Plantago major* L.) in order to cover all major clades of the whole species pool (Figure S1). The number of species in each clade approximately reflects relative species richness of clades of the species pool of the case study in species‐rich grasslands. Then, we simulated 1,000 communities using all the 10 species and let their proportions randomly vary. Phylogenetic richness, divergence, and regularity were estimated for each simulated community. (b) Faith's PD particularly increased with increasing proportion of *R. repens* (i.e., the relatively most phylogenetically distant species compared to the rest). Distant branches contribute more to phylogenetic richness as they are longer, suggesting that increase in their weight (reflecting species proportion in a community) also increases phylogenetic richness of a community. (c) Histogram of simulated MPD values. MPD of a community when all four clades are equally abundant (*p*_monocots_ = *p*_Ranunculales_ = *p*_superrosids_ = *p*_superasterids_) is indicated. (d) Histogram of simulated VPD values. VPD of a community when each species has same proportion (i.e., proportion of each clade is equivalent to its relative species richness) is indicated

The phylogenetic categorical approach cannot rely on phylogenetic distances, but we can include information about how clades are represented in a community (presence and relative abundance) to estimate its phylogenetic diversity. Consider a simple example phylogeny of 10 species (Figure 1a), which covers all major clades of the whole species pool of our first case study (Figure S1). We simulated 1,000 communities where these 10 species occurred, but we let their proportions in a community randomly vary. For each community, we estimated phylogenetic richness, divergence, and regularity (sensu Tucker et al., 2017) using a leading metric of each dimension (see Methods for more information). Visual inspection of phylogeny‐based measures showed several interesting features. Phylogenetic richness increased with increasing proportion of the most distantly related species (*Ranunculus repens* in this case) in comparison with the rest of the species in the community (Figure 1b). Phylogenetic divergence was relatively high when all defined clades (i.e., monocots, Ranunculales, superrosids, and superasterids) had equal proportions (Figure 1c). Finally, phylogenetic regularity was relatively high (i.e., the variance of phylogenetic distances was low) when the defined clades had proportions proportional to their relative species richness in the species pool (Figure 1d).

Based on the conclusions from the conceptual example described above, we propose here three alternative measures, the clade indices that do not require dated phylogenies for their computation, but instead they utilize information about clade proportions in a community and species richness of a community or defined clades (Table 2). We assessed their fit accuracy for leading phylogeny‐based measures of the three dimensions of phylogenetic diversity: richness, divergence, and regularity (sensu Tucker et al., 2017). To do so, we examined the performance of the proposed clade indices in two case studies, firstly with a dataset with a purpose‐built phylogeny (sensu Li et al., 2019) consisting of relatively small number of taxa in the species pool and second dataset with a synthesis‐based phylogeny (sensu Li et al., 2019) consisting of relatively large number of taxa in the species pool. In this first case study, we also examined what clade resolution (at the super‐order, order, and family level) for the clade index definition is the most suitable in terms of fit accuracy for phylogeny‐based measures. Secondly, we used simulated community matrices based on a megaphylogeny of 31,389 vascular plants (Qian & Jin, 2016) to demonstrate how the clade indices perform at various phylogenetic scales (Graham, Storch, & Machac, 2018), at different species pool sizes and along a species richness gradient.

**Table 2:** Summary of the proposed clade indices

Index	Equation	Treatment	Rationale
(a) Clade richness	$\log\left(S\right)+3\cdot \sum_{i=1}^{k}\frac{p_{i}}{{\text{CR}}_{i}}$	Species‐rich clades are penalized as they get lower weight proportional to their clade richness. Higher proportions of species‐poor clades increase the clade richness index values	Species from species‐poor clades have higher probability to be relatively phylogenetically distant to the rest of a community and their increasing proportion increases phylogenetic richness of a community (Figure 1b)
(b) Clade divergence	$1-\sum_{i=1}^{k}{\left(p_{i}-\frac{1}{{\text{CR}}_{\text{SP}}}\right)}^{2}$	Larger deviations from optimal proportions (i.e., 1/number of defined clades in the whole species pool) decrease the value of the clade divergence index. Scales from 0 to 1	Phylogenetic divergence tends to be close to its peak when a community consists of all clades of a species pool and their proportions are equal (Figure 1c)
(c) Clade regularity	$1-\sum_{i=1}^{k}{\left(p_{i}-\frac{{\text{CR}}_{i}}{S_{\text{SP}}}\right)}^{2}$	Larger deviations from the optimal proportions (i.e., clade species richness/total species pool richness) decrease the value of the clade regularity index. Scales from 0 to 1	Phylogenetic regularity tends to be close to its peak (the lowest VPD) when a community consists of all clades of a species pool and their proportions are proportional to their relative clade richness given a species pool (Figure 1d)

Note

*S* = species richness of a plot; *p_i_* = proportion of the *i*th clade in a plot; CR*_i_* = species richness of the *i*th clade in the whole species pool (all species in the dataset); CR_SP_ = the number of all defined clades in the whole species pool; *S*_SP_ = species richness of the whole species pool.

## 2 MATERIALS AND METHODS

### 2.1 Data collecting

The focus of the case studies was on herbaceous terrestrial systems. First, we used data from species‐rich grasslands located in two Protected Landscape Areas on the border between the Czech Republic and Slovakia: Beskydy Mountains (N 49.45°, E 18.33°) and White Carpathian Mountains (N 48.97°, E 17.82°). We collected vegetation records in 240 permanent plots (1 × 1 m in size) in 12 long‐term management experiments (hereafter exclosures) at six localities (Table S1) in 2013. Community data included 171 plant species. Second, we assembled vegetation plots from a stratified dataset (for detailed information, see Chytrý, Pyšek, Tichý, Knollová, & Danihelka, 2005) extracted from the Czech National Phytosociological Database (hereafter CNPD; Chytrý & Rafajová, 2003). This dataset included 16,542 plots and 1,608 species and covered 26 Central European herbaceous habitats (see Table S2 for a habitat classification). We limited our analysis to herbaceous angiosperms that dominate all systems used in this study. In the grassland dataset, tree taxa were omitted in the initial phase of the vegetation recording, but this most likely did not affect estimation of phylogenetic diversity as we found only a few tree seedlings in a few plots. We deleted Pteridophyta from both datasets, whereas gymnosperms did not occur in any dataset.

### 2.2 Phylogenetic inference and molecular dating

Prior to the phylogenetic analysis, we checked species lists and edited some species names in order to follow the NCBI nomenclature. For the species‐rich grasslands, we constructed a molecular‐based phylogeny for our 171 species using 20 orthologous loci downloaded from GenBank (Benson et al., 2017) via an online tool OneTwoTree (Drori et al., 2018). We used *Piper nigrum* L. from the Magnoliids group (a sister clade to clades occurring in our dataset; APG IV, 2016) as an out‐group. Due to missing sequence data, we replaced *Potentilla heptaphylla* L. with a relatively close congener *Potentilla crantzii* (Crantz) Beck ex Fritsch (Dobeš, Rossa, Paule, & Hülber, 2013) that had available DNA data. Sequences were aligned using a fast option (FFT‐NS‐2) in MAFFT (Katoh & Standley, 2013) under default settings available at the OneTwoTree website (6mer pairwise alignment method). The alignment was then cured using the Gblocks online tool (under less stringent selection settings; Castresana, 2000).

We constructed the dated tree using BEAST version 1.10.4 (Suchard et al., 2018) in the CIPRES portal (Miller, Pfeiffer, & Schwartz, 2010). To do so, we manually set constraints according to the APG IV angiosperm phylogeny (APG IV, 2016) and set the uncorrelated relaxed clock as a clock model, Yule process as a speciation model and GTR+G+I (with four gamma categories) as a nucleotide substitution model. To translate genetic distances into absolute times, we exploited the TimeTree database (Kumar, Stecher, Suleski, & Hedges, 2017) and set several time priors with normally distributed errors (median and standard deviation computed from all studies available in the TimeTree database reporting a given divergence time estimate). We performed three independent runs (with different starting seeds) for 100 million generations each. Finally, we checked convergence in Tracer v1.7.1 (Rambaut, Drummond, Xie, Baele, & Suchard, 2018) and combined all runs (10% generations as a burn‐in). The dated maximum clade credibility tree (Figure S1) was sampled from 30,000 trees (10% trees as a burn‐in).

For the species in the dataset from the CNPD, we extracted species phylogeny from the dated supertree of the European flora (Durka & Michalski, 2012) and followed their nomenclature.

### 2.3 Phylogenetic diversity dimensions and metrics

We applied the framework of Tucker et al. (2017) and selected three leading metrics describing three phylogenetic diversity dimensions: richness, divergence, and regularity (Table 1). Faith's PD (Faith, 1992) describes the amount of evolutionary history across species (sum of branch lengths) and is a leading measure of phylogenetic richness. Mean phylogenetic distance between each pair of species (MPD; Webb et al., 2002) is a leading measure of phylogenetic divergence. Variation of pairwise phylogenetic distances between each pair of species (VPD; Clarke & Warwick, 2001) is a leading measure of phylogenetic regularity (lower variation indicates higher regularity). We also identified species richness in each plot.

According to Vellend et al. (2011), one can distinguish two qualitatively different types of phylogenetic diversity indices. Faith's PD, MPD, and VPD are type II metrics which are calculated using a subset phylogeny of a focal subset of species (e.g., a vegetation plot). Type I indices are based on the whole species pool phylogeny; each species has its distinctness score calculated. These scores are then used to calculate a phylogenetic diversity measure of a plot (for example, summed evolutionary distinctiveness; Redding & Mooers, 2006). However, type I indices are highly correlated with Faith's PD (Vellend et al., 2011), suggesting they are closely related to the phylogenetic richness dimension, and so we did not consider them. We calculated indices using functions (*pd* and *mpd*) from the *picante* package (Kembel et al., 2010). To compute VPD, we modified the *mpd* function to calculate the variation of pairwise phylogenetic distances (not the mean as in the original function). All metrics were abundance weighted by percentage cover. To calculate abundance‐weighted Faith's PD (Barker, 2002), we used the R function of Swenson (2014).

### 2.4 Definition of the clade indices

Species affiliation to a clade was based on the recent APG IV classification (APG IV, 2016). The proposed clade indices are summarized in Table 2. They all need information about clade proportions in a community (e.g., relative cover, biomass or abundances). The key idea behind the clade richness index is to penalize proportions of species‐rich clades (by reverse clade species richness) because species from species‐rich clades are unlikely to be relatively distantly related to the rest of co‐occurring species in a community. By chance, more species from a species‐rich clade can occur in a community, which would decrease phylogenetic richness as these species are relatively closely related. Species richness can be a very good indicator of phylogenetic richness by its own (Swenson, 2014; Vellend et al., 2011); hence, it is useful to include it in the equation (Table 2a). For phylogenetic divergence, when clades are equally abundant in a community, phylogenetic divergence is close to its peak (Figure 1c). Thus, any deviations from these equal proportions should decrease phylogenetic divergence (Table 2b). For instance, if all clades are present and have equal (i.e., optimal) proportions, the clade divergence index equals one. Finally, the clade regularity index has a similar computation to the clade divergence index, but the optimal proportions are proportional to the relative clade species richness (Table 2c). An R script for computation of the clade indices is stored in the supplemental dataset (https://data.mendeley.com/datasets/gbv472pxsb/1).

### 2.5 Performance of the clade indices: case studies

We did all statistical analyses and data simulations in R version 3.6.0. (R Core Team, 2019). Faith's PD was square‐root transformed, and VPD was log‐transformed prior to the analysis. First, we examined how the different phylogenetic resolutions affect values of the clade indices and their correlations with phylogeny‐based indices. To do so, we used the grassland dataset and tested three clade resolutions: (a) super‐order level (monocots, Ranunculales, superrosids, and superasterids), (b) order (based on affiliation to 20 orders), and (c) family (based on affiliation to 32 families). We calculated the clade indices and assessed their fit of phylogeny‐based indices using linear models (the *lm* function in R) and estimated *R* ^2^ values. We also checked for the significance of quadratic terms. In the case of phylogenetic regularity, we used generalized least squares models (the *gls* function, *nlme* package; Pinheiro, Bates, DebRoy, & Sarkar, 2019) to acknowledge heteroscedasticity (using the exponential variance class, *varExp*), which we detected during the model diagnostics.

### 2.6 Performance of the clade indices: simulated datasets

Simulation workflow was specifically designed to cover several aspects that can affect phylogenetic diversity estimation, that is, taxon sampling (Park et al., 2018), the number of taxa included in the regional phylogeny (Jantzen et al., 2019) or species richness of a community (Sandel, 2018; Swenson, 2014). Thus, these factors could also affect fit accuracy of the clade indices for all dimensions of phylogenetic diversity. The simulation workflow is summarized in Figure S2. Simulation was based on a megaphylogeny of vascular plants (Zanne et al., 2014, updated by Qian & Jin, 2016). We set three phylogenetic scales: vascular plants, angiosperms, and superasterids. For each phylogenetic scale, we set three species pool sizes: 2,000, 500, and 250 species. These species pools were created by randomly assigning species from a given phylogeny (vascular plants, angiosperms, or superasterids). For each combination of phylogenetic scale and species pool size, we generated community matrices under several species richness ranges: 10–160, 10–80, 10–40, 10–20, 5–10, and 2–5 species per community. For each species richness range, we generated 50 community matrices with 240 sites (same data size as in the grassland case study). Species proportions in communities were random but their sums were always one. In total, we generated 2,700 unique species pools with 2,700 unique corresponding community matrices (900 for each phylogenetic scale).

For each community matrix, we computed both phylogeny‐based metrics (Faith's PD, MPD and VPD) and the clade indices. Then, we performed linear models with phylogeny‐based metrics as response variables and clade indices as explanatory variables and extracted each models *R* ^2^ values. Faith's PD was always square‐root transformed; VPD was always log‐transformed. To assess the importance of all determinants potentially affecting the relationship between phylogeny‐based metrics and the clade indices, we calculated relative variances of *R* ^2^ values attributed to either phylogenetic scale, species pool size or species richness range using the *VarCorr* function (*nlme* package, Pinheiro et al., 2019). The determinants were hierarchically structured in the model random‐effect formula (phylogenetic scale/species pool size/species richness range).

## 3 RESULTS

**Figure 2:**
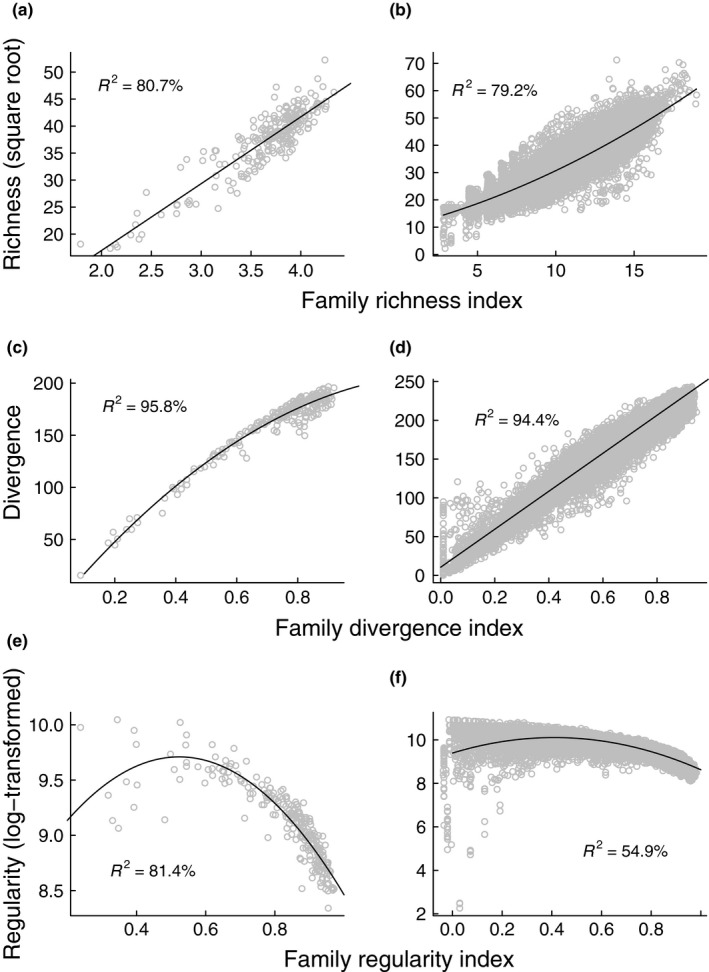
Fit accuracy of the clade indices in the case studies: species‐rich grasslands (a,c,e) and the Czech National Phytosociological Database (b,d,f). (a,b) The phylogenetic richness dimension (described by Faith's PD). (c,d) The phylogenetic divergence dimension (described by MPD). (e,f) The phylogenetic regularity dimension (described by VPD). Number of plots: species‐rich grasslands = 240, the Czech National Phytosociological Database = 16,542

For all phylogenetic diversity dimensions, fit accuracy of the clade indices increased with fineness of phylogenetic resolution in species‐rich grasslands (Table S3); hence, we present here the clade indices based on the resolution at the family level in all case studies and simulated communities. For phylogenetic richness and divergence, the fit was reasonably high and similar in both case studies (Figure 2a–d), and in all herbaceous habitats (the CNPD dataset) when fitted separately (Figures S3 and S4). For phylogenetic regularity, fit accuracy increased with increasing values of the family regularity index (Figure 2e,f) as the relationship was accompanied with decreasing heteroscedasticity. Models are summarized in Table S4.

Heteroscedasticity was mainly apparent in the CNPD dataset (Figure 2f) at the left end of the clade regularity index gradient (a range of values from 0.0 to 0.2, approximately). This was partly the reason of habitat dependency because the clade regularity index showed changeable fit accuracy across habitats (Table S5), and the heteroscedasticity issues at the left end were mainly caused by several habitats (Figure S5), such as C1 (surface standing waters) or C2 (surface running waters). Partly, the broader taxon sampling in the CNPD phylogeny was the reason for a large range of VPD values (approximately, three times higher than in species‐rich grasslands). The variance of VPD values was largest at the left end, where the clade regularity index explained VPD less accurately (Figure 2f). Nevertheless, *R* ^2^ rapidly increased (72.3%) when we only included plots with the clade regularity index higher than 0.2 (93.8% of all plots). For phylogenetic richness and divergence, fit accuracy across habitats was usually similar (more than 70% for phylogenetic richness and more than 90% for phylogenetic divergence) with several exceptions with lower *R* ^2^ values, such as H2 (screes) or E4 (alpine and subalpine grasslands). Fit accuracy in all habitats is given in Table S5.

**Figure 3:**
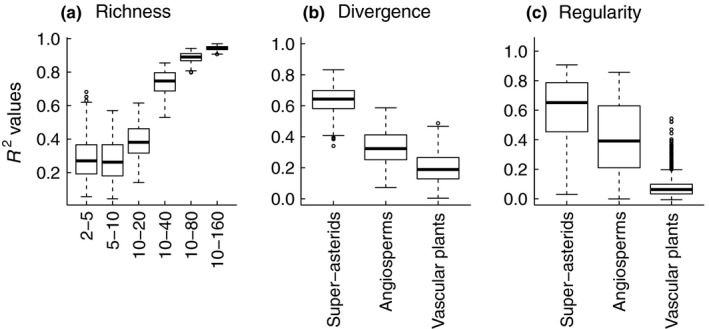
Major determinants of fit accuracy of the clade indices in simulated communities (species richness range for phylogenetic richness and phylogenetic scale for divergence and regularity; Table 3). (a) Phylogenetic richness: Faith's PD against family richness index in different species richness ranges, (b) phylogenetic divergence: MPD against family divergence index at different phylogenetic scales, and (c) phylogenetic regularity: VPD against family regularity index at different phylogenetic scales

Simulated datasets revealed that species richness range was the most important determinant of fit accuracy of the clade richness index, while phylogenetic scale mainly affected fit accuracy of the clade divergence and regularity indices, followed by species richness (Table 3). Species pool size did not influence fit accuracy for any phylogenetic diversity dimension (Table 3). For phylogenetic richness and regularity, *R* ^2^ values increased with increasing species richness range (Figure 3a, Figure S6d,e). For phylogenetic divergence and regularity, fit accuracy increased with decreasing phylogenetic scale, *R* ^2^ was highest in community matrices sampled from the phylogeny of superasterids (Figure 3b,c), while the clade indices for these two dimensions were less reliable at the largest phylogenetic scale, that is, vascular plants (Figure 3b,c). At smaller phylogenetic scales (angiosperms and superasterids), fit accuracy for phylogenetic regularity also increased with increasing species richness range (Figure S6d,e), but this was not the case when we sampled community matrices using the whole phylogeny of vascular plants, that is, the largest phylogenetic scale considered (Figure S6f). Interestingly, the *R* ^2^ values for phylogenetic divergence were generally lower compared with the case studies where the family divergence index provided exceptional fit accuracy (95.8% and 94.4%), while the *R* ^2^ values very rarely exceeded 80% in simulated communities and the average was only 39%. In general, fit accuracy tended to be lower in simulated communities with low species richness, suggesting unreliability of the clade indices as surrogates of phylogenetic diversity in species‐poor habitats or at very small spatial scales.

**Table 3:** Variance components of the hierarchically structured factors used for generating artificial communities

Factor	Richness	Divergence	Regularity
Phylogenetic scale	<0.1	62.1	51.5
Species pool size	<0.1	<0.1	<0.1
Species richness range	86.8	20.2	33.7
Residual	13.2	17.6	14.8

Note

Values (%) depict relative variance of *R* ^2^ values (fit accuracy of the proposed clade indices for all dimensions of phylogenetic diversity) attributed to a factor. Phylogenetic scale reflects a clade used for species pool generating (vascular plants, angiosperms, or superasterids). A megaphylogeny of vascular plants was taken from Qian and Jin (2016). Species pool size indicates the number of species in a regional phylogeny (2,000, 500, or 250). Species richness range indicates a range restricting the number of species in artificial communities (2–5, 5–10, 10–20, 10–40, 10–80, and 10–160). In total, 2,700 unique species pools and corresponding community matrices were generated.

## 4 DISCUSSION

We have shown that simple taxonomic coding at the family level can be used to accurately indicate phylogenetic diversity in plant communities. We propose three simple surrogates of phylogenetic diversity, the clade indices, which only require information about species affiliation to a clade and clade proportions in samples, while phylogenetic distances among species are not necessary (Table 2). Our indices provided an accurate fit to leading phylogenetic diversity metrics as shown for our two case studies: 1 × 1 m plots from species‐rich grasslands and phytosociological relevés of various sizes from 26 Central European herbaceous habitats (Figure 2, Figures [Link], [Link], [Link]). Our simulations indicate that the clade indices are highly correlated with phylogeny‐based metrics in samples with 10 or more species (richness and regularity dimensions) and in species pools sampled from relatively smaller phylogenetic scales, such as angiosperms (estimated root age around 181 MYA, Kumar et al., 2017) or hierarchically lower clades (divergence and regularity dimensions; Figure 3, Figure S6). Using phylogenetic trees definitely provides the most detailed information about phylogenetic patterns in communities, however, due to the lack of resources (time or money) our proposed method can be used as a reliable proxy of phylogeny‐based measures.

Clade indices can be used to simplify some aspects of the whole workflow behind phylogenetic diversity estimation. First, it enables the speeding‐up of vegetation recording for any project involving a phylogenetic framework as species need to be correctly determined only at the higher taxonomic level (but note that the clade richness index requires species richness of a community for its computation). This is a welcome simplification, especially when dealing with hard to determine taxa. Second, phylogenetic diversity estimation using the clade indices requires less effort, expertise, and cost, as there is no need to obtain molecular data, performs a phylogenetic analysis and molecular dating (the latter is not necessary when phylograms are used, i.e., branch lengths in units substitutions per site; but see Jantzen et al., 2019 for discussion of how phylogenetic diversity measures can be affected by using either phylograms or dated phylogenies). As discussed in Li et al. (2019), researchers have to decide what markers (Which genes to select?) and methods to use (Alignment method? Model of evolution? Maximum likelihood or Bayesian inference framework? What fossil constraints for molecular dating?). All these difficult methodological decisions can be also avoided using phylogenies pruned from supertrees, for example, Daphne (Durka & Michalski, 2012) or the Open Tree of Life (Hinchliff et al., 2015), which have been shown to provide estimates of phylogenetic diversity well correlated to those derived from purpose‐built phylogenies (Li et al., 2019) and, additionally, have broader taxon sampling coverage that is important to correctly estimate phylogenetic diversity (Jantzen et al., 2019; Park et al., 2018). On the other hand, for many taxonomic groups, supertrees are poorly sampled and unavailable (e.g., Daphne covers only a part of the European flora) or do not include branch lengths (Open Tree of Life) that need to be additionally calculated (Li et al., 2019).

The larger CNPD phylogeny with a broader taxonomic sampling created an almost three times larger range of VPD values in the CNPD compared to the grassland dataset. Due to this issue, we particularly encountered problems with heteroscedasticity. In species‐ and clade‐poor habitats, the fit was generally poor (Table S5, Figure S5). For example, water habitats (C1 and C2) or carr and fen scrubs (F9.2) usually host specialized species from very few clades (e.g., Alismataceae or Salicaceae, respectively). Phylogenetic regularity of communities in these habitats will be highly dependent on the presence of other arms from the angiosperm radiation, as more distantly related lineages decrease phylogeny balance more than closely related ones, that is, the degree to which branch points define subgroups of equal size (Heard, 1992). Vellend et al. (2011) provide relevant discussion of the effect of tree imbalance on phylogenetic diversity assessment. Thus, we suggest using the clade regularity index in relatively species‐rich communities where its values are higher than 0.2, and recommend the estimation of phylogenetic regularity using phylogeny‐based measures in communities where the clade regularity index ranges from 0 to 0.2. For phylogenetic richness and divergence, fit accuracy of the clade indices was consistent across all the studied habitats (Table S5, Figures 2, S3 and S4) and was, therefore, not affected by taxon sampling in the case studies.

Simulated community matrices highlighted the effect of species richness and phylogenetic scale on fit accuracy of the clade indices (Table 3). Species richness affects the values of phylogeny‐based measures either directly or indirectly through shaping their range of possible values (Swenson, 2014; Vellend et al., 2011). In species‐poor communities, the range of possible values of phylogeny‐based measures was relatively high (Figure S7), and the clade indices (richness and regularity) tracked this variance less accurately (Figure 3a, Figure S6d,e). This suggests lower reliability of our method at very small spatial scales where plots consist of few species (<10). In contrast to species richness, increasing phylogenetic scale increases the possible range of phylogenetic distances because more distantly related species can occur in a community. As expected, fit accuracy for phylogenetic divergence and regularity was better at smaller phylogenetic scales (superasterids and angiosperms). For phylogenetic divergence, we observed a disparity in fit accuracy between case studies (substantial *R* ^2^ values) and simulated community matrices (moderate *R* ^2^ values). This could be probably attributed to the simulation protocol. Simulated community matrices were completely random in terms of species selection and species proportions, which does not reflect nonrandom assembly processes in nature. Sometimes, fit accuracy was greatly improved by log‐transforming MPD values, but this mainly depended on the generated community matrix and we did not find consistent improvements after the log‐transformation when comparing phylogenetic scales or species richness ranges. On the other hand, our case studies indicate that the phylogenetic divergence index is a very precise surrogate of MPD for real vegetation data (Figure 2c,d). In summary, the results suggest we should expect tight correlations between the clade indices and all dimensions of phylogenetic diversity in angiosperm‐dominated habitats where samples have more than 10 species.

Community and phylogenetic data influence the computation, behavior, or type I and II errors of phylogenetic diversity estimates (Cadotte et al., 2010; Miller, Farine, & Trisos, 2017; Tucker et al., 2017; Vellend et al., 2011). Certain features need to be considered when using clade proportions as an indicator of phylogenetic diversity. First, an outcome is dependent on the type of community data (presence/absence versus abundance weighted). Since the clade indices proposed here require information about relative abundances, they are not useful for presence/absence data. Second, phylogenetic diversity is expected to provide additional information than species richness and diversity. Usually, phylogenetic diversity metrics are positively correlated with species richness (Faith's PD) or at least the range of their possible values declines as the number of species increases (MPD; Swenson, 2014). As expected, clade indices showed the same decline of possible values with increasing species richness (Figure S7). To account for possible bias due to species richness variation, null models or rarefaction is recommended (Miller et al., 2017; Sandel, 2018; Swenson, 2014). Both tools can be used to treat species richness‐dependence of clade indices. Finally, phylogenetic resolution influences the performance of the clade‐based approach. As expected, our results indicate that increasing fineness of phylogenetic resolution increases the tightness of the relationship between phylogeny‐based measures and clade indices (Table S3). This agrees with case studies and simulated phylogenies that showed a lower impact of the lack of resolution or poorly estimated branch lengths at more recent nodes on phylogenetic diversity (Allen et al., 2019; Swenson, 2009). Naturally, our method can be prone to taxonomic errors as it assumes proper species assignments to defined taxonomic groups.

Our goal was to show the link between clade composition and phylogenetic diversity. Our results suggest that the clade indices proposed here, which are based on taxonomic resolution at the family level, are a good indicator of all phylogenetic diversity dimensions in angiosperm‐dominated habitats with 10 and more species per sampling unit (e.g., 1 m^2^ or larger plots in grasslands). Even though this study focused on vascular plants, our results should generalize to any taxonomic group with a well‐developed taxonomic classification supported by molecular data. In general, if a taxonomic classification of a group reflects current molecular phylogenies we should expect close correlations between taxonomy‐based metrics (e.g., this study, Warwick & Clarke, 1995) and molecular‐based phylogenetic metrics. Our approach has a potential in studies working with a lot of taxa when phylogenetic reconstruction might be very time‐ and money‐consuming.

## CONFLICT OF INTEREST

None declared.

## AUTHOR CONTRIBUTIONS

MB and PM conceived the ideas, designed the study, and analyzed the data. MB conducted phylogenetic analysis and wrote the manuscript with help from RJP and MD. All authors discussed the results, contributed critically to the drafts and gave final approval for publication.

## Supporting information

Figure S1

Figure S2

Figure S3

Figure S4

Figure S5

Figure S6

Figure S7

Supplementary Material

## Data Availability

All data supporting the results (accession numbers, alignment matrices, BEAST.xml file, phylogenetic trees, plot data, species lists, and simulation results) are archived in the Mendeley Data depository (https://data.mendeley.com/datasets/gbv472pxsb/1).
