# Supplementary figures and images for: Clade composition of a plant community indicates its phylogenetic diversity
Source: Ecol Evol. 2020 Mar 13;10(8):3747–57. doi: 10.1002/ece3.6170 (PMC7160181; doi:10.1002/ece3.6170)

1

1

0.4725

Super-asterids  
(78 species)

## Monocots (38 species)

## Ranunculales (5 species)

## Super-rosids (50 species)

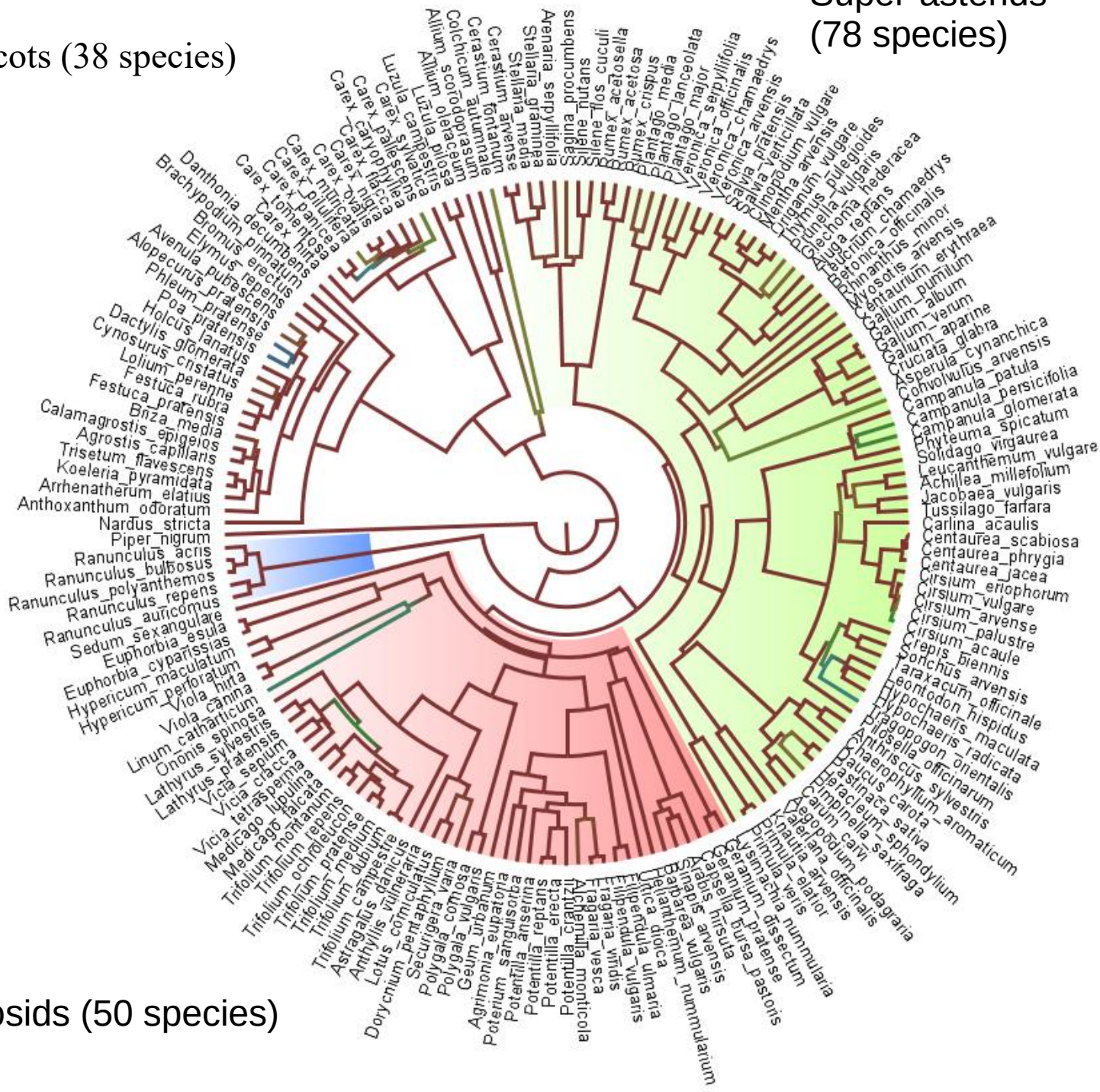

Supplement: Supplementary file 1 — Figure S1 [file ECE3-10-3747-s001.pdf]

Richness (Faith's PD, square root)

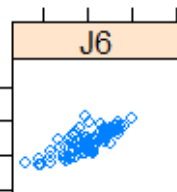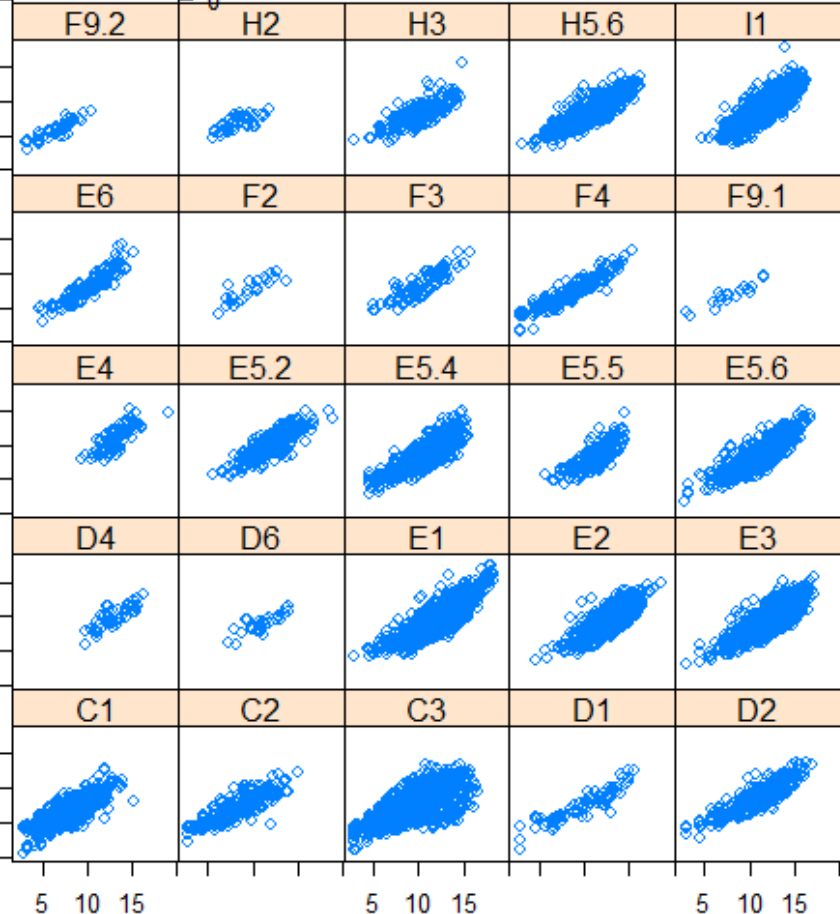

Family richness index

Supplement: Supplementary file 3 — Figure S3 [file ECE3-10-3747-s003.pdf]

Divergence (MPD)

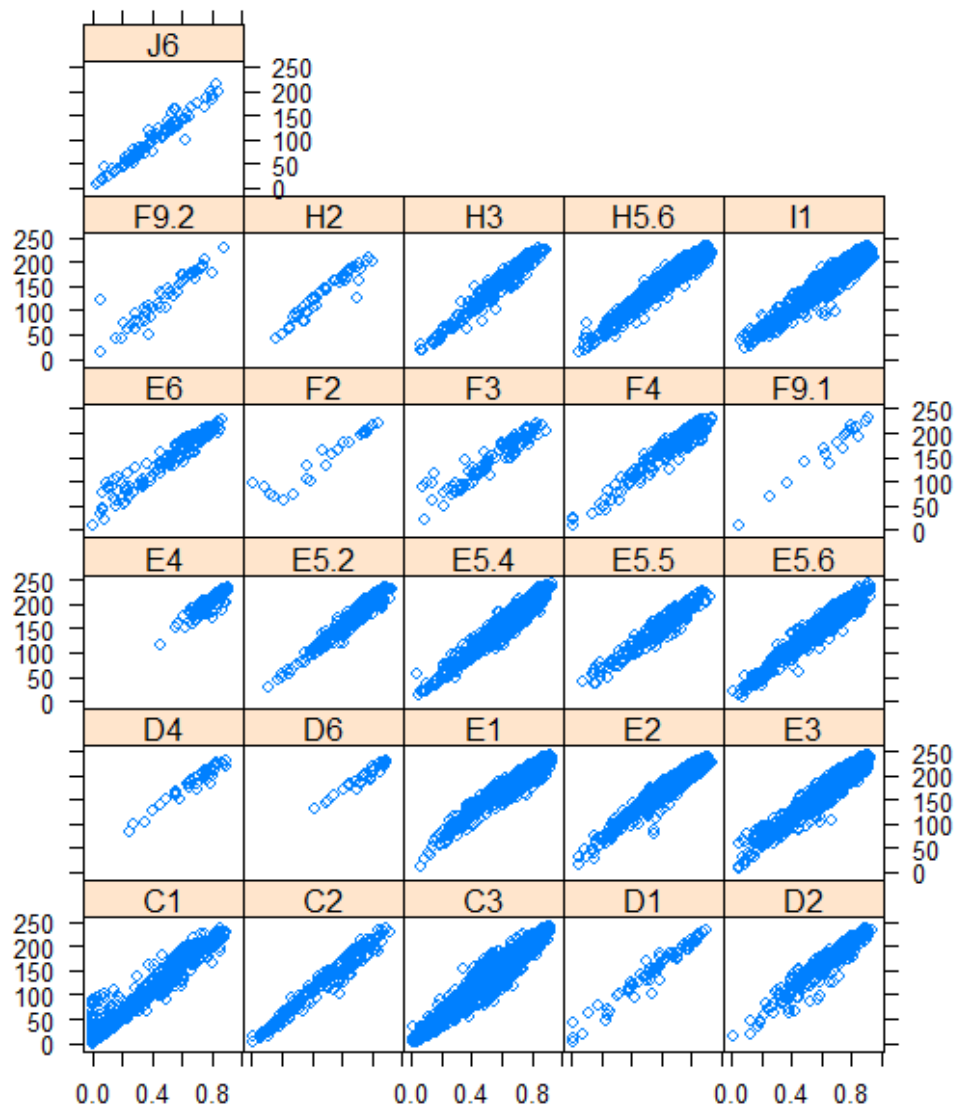

Family divergence index

Supplement: Supplementary file 4 — Figure S4 [file ECE3-10-3747-s004.pdf]

Regularity (VPD, log-transformed)

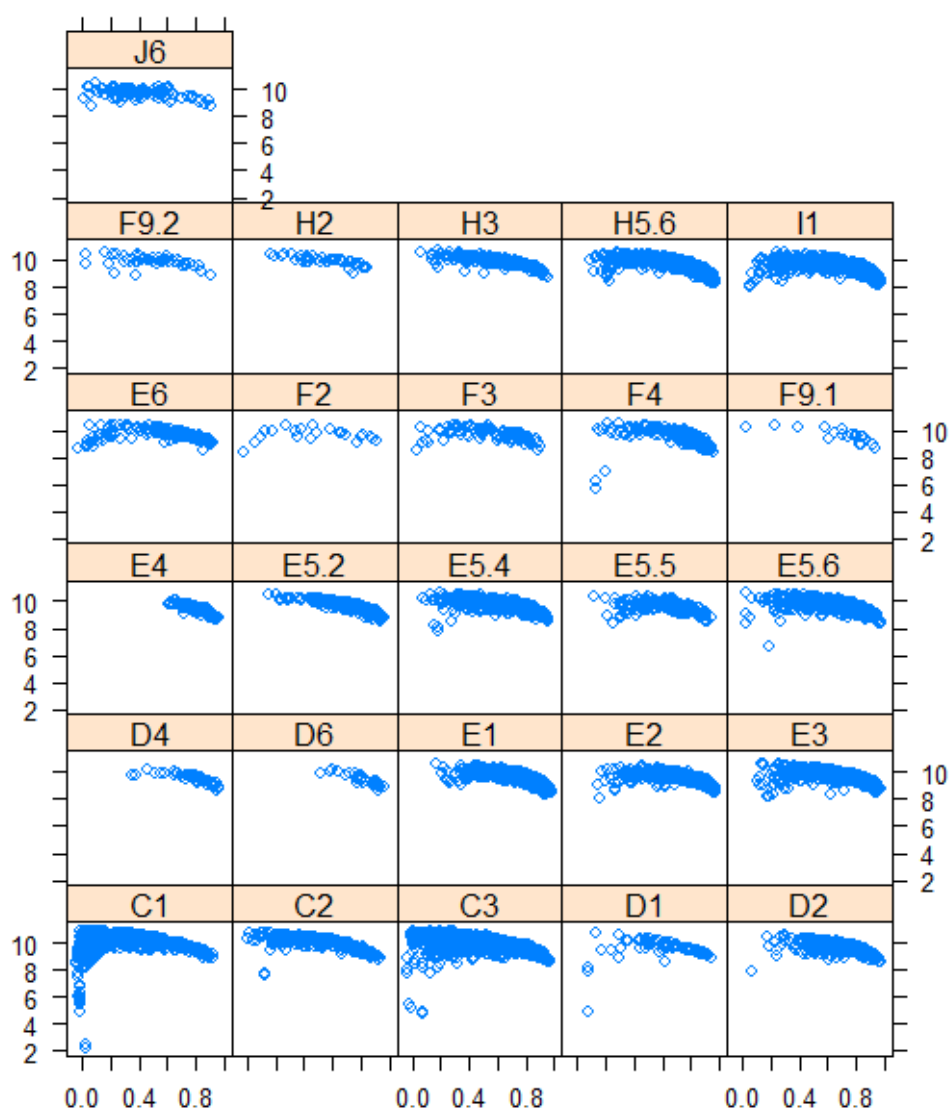

Family regularity index

Supplement: Supplementary file 5 — Figure S5 [file ECE3-10-3747-s005.pdf]
