# Supplementary material for: Clade composition of a plant community indicates its phylogenetic diversity
Source: Ecol Evol. 2020 Mar 13;10(8):3747–57. doi: 10.1002/ece3.6170 (PMC7160181; doi:10.1002/ece3.6170)
Supplement: Supplementary file 2 — Figure S2 [file ECE3-10-3747-s002.pdf]

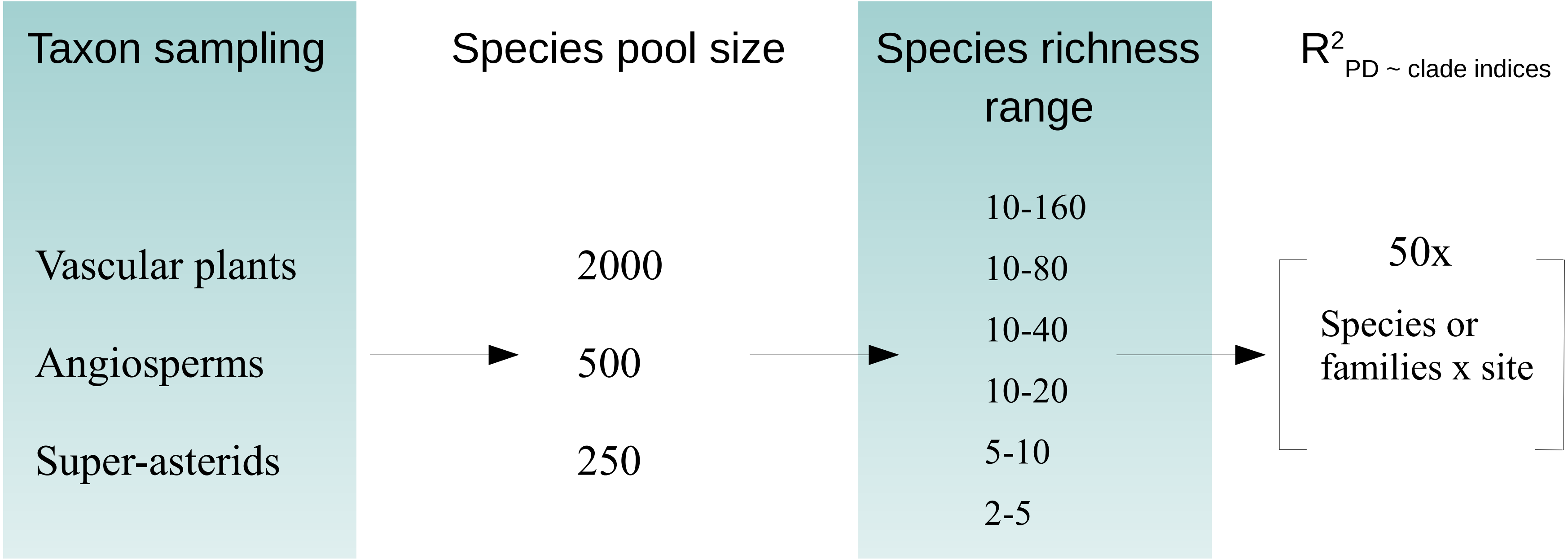
Taxon sampling
Species pool size
Species richness range
$R^2_{PD \sim clade\ indices}$
Vascular plants
Angiosperms
Super-asterids
2000
500
250
10-160
10-80
10-40
10-20
5-10
2-5
50x
Species or families x site
