# Supplementary material for: Clade composition of a plant community indicates its phylogenetic diversity
Source: Ecol Evol. 2020 Mar 13;10(8):3747–57. doi: 10.1002/ece3.6170 (PMC7160181; doi:10.1002/ece3.6170)
Supplement: Supplementary file 8 — Supplementary Material [file ECE3-10-3747-s008.docx]

**APPENDICES**

**Table S1** Location, description, and GPS coordinate of the 12 exclosures (long-term experiments) in species-rich grasslands.

**Table S2** Summary of herbaceous habitats used in this study.

**Table S3** Comparison of coefficients of determination (R^2^) when using different phylogenetic resolution for the definition of the clade indices.

**Table S4** Summary of models presented in Figure 2 (case studies).

**Table S5** R^2^ values (%) of the clade indices fitting all three dimensions of phylogenetic diversity separately in each herbaceous habitat in the CNPD.

**Figure S1** Phylogeny of 171 plant species occurring in 12 exclosures (long-term experiments) in species-rich grasslands.

**Figure S2** Simulation workflow.

**Figure S3** Phylogenetic richness (Faith’s PD) plotted against the clade richness index separately in each herbaceous habitat (Czech National Phytosociological Database dataset).

**Figure S4** Phylogenetic divergence (MPD) plotted against clade divergence index separately in each herbaceous habitat (Czech National Phytosociological Database dataset).

**Figure S5** Phylogenetic regularity (VPD) plotted against clade regularity index separately in each herbaceous habitat (Czech National Phytosociological Database dataset).

**Figure S6** Fit accuracy in simulated community matrices for phylogenetic divergence and regularity along the gradient of species richness ranges at different phylogenetic scales.

**Figure S7** Dependence of phylogenetic diversity metrics and clade indices on species richness.

**Table S1** Location, description, and GPS coordinate of the 12 exclosures (long-term experiments) in species-rich grasslands.

| **a) Beskydy Protected Landscape Area**  Experimental exclosures established at three localities in 2006 | | | |  | |
| --- | --- | --- | --- | --- | --- |
| Site |  | Description | Exclosure | Latitude | Longitude |
| Půlčín |  | Alliance *Arrhenatherion elatioris*: mesic meadows with dominance of *Agrostis capillaris* L. | A | N 49.227491° | E 18.079431° |
|  |  |  | B | N 49.22571° | E 18.078193° |
| Kýchová |  | Alliance *Bromion erecti*: broad-leaved dry grassland with dominance of *Brachypodium pinnatum* (L.) P. Beauv. | C | N 49.291101° | E 18.132017° |
|  |  |  | D | N 49.291847° | E 18.133777° |
| Losový |  | Alliance *Bromion erecti*: broad-leaved dry grassland with dominance of *Brachypodium pinnatum* | E | N 49.317729° | E 18.094185° |
|  |  |  | F | N 49.317549° | E 18.102602° |
| **b) White Carpathians Protected Landscape Area**  Experimental exclosures established at three localities in 2004 | | | | | |
| Brumov |  | Alliance *Bromion erecti*: broad-leaved dry grassland with dominance of *Brachypodium pinnatum* | G | N 49.097265° | E 18.029639° |
|  |  |  | H | N 49.098787° | E 18.028752° |
| Lopeník |  | Alliance *Cynosurion cristati*: mesic pasture with dominance of *Festuca rubra* L. | I | N 48.936828° | E 17.801392° |
|  |  |  | J | N 48.937219° | E 17.800780° |
| Suchov |  | Alliance *Violion caninae*: submontane grassland with abundant *Nardus stricta* L. | K | N 48.896432° | E 17.580845° |
|  |  |  | L | N 48.896876° | E 17.580496° |

**Table S2** Herbaceous habitats used in this study (extracted from Chytrý & Rafajová, 2003). Abbreviations (Code) follow the EUNIS habitat classification (Davies & Moess 2003).

| Habitat | Code | n | Habitat | Code | n |
| --- | --- | --- | --- | --- | --- |
| Surface standing waters | C1 | 1028 | Subalpine moist or wet tall-herb and fern habitats | E5.5 | 218 |
| Surface running waters | C2 | 254 | Anthropogenic forb-rich habitats | E5.6 | 799 |
| Littoral zone of inland surface waterbodies | C3 | 2891 | Inland saline grass and herb-dominated habitats | E6 | 151 |
| Raised and blanket bogs | D1 | 75 | Arctic, alpine and subalpine scrub habitats | F2 | 24 |
| Valley mires, poor fens and transition mires | D2 | 375 | Temperate and mediterraneo-montane scrub habitats | F3 | 102 |
| Base-rich fens | D4 | 49 | Temperate shrub heathland | F4 | 228 |
| Inland saline and brackish marshes and reedbeds | D6 | 32 | Riverine and lakeshore [Salix] scrub | F9.1 | 20 |
| Dry grasslands | E1 | 2508 | [Salix] carr and fen scrub | F9.2 | 48 |
| Mesic grasslands | E2 | 1698 | Screes | H2 | 50 |
| Seasonally wet and wet grasslands | E3 | 2251 | Inland cliffs, rock pavements, and outcrops | H3 | 236 |
| Alpine and subalpine grasslands | E4 | 94 | Trampled areas | H5.6 | 777 |
| Thermophile woodland fringes | E5.2 | 369 | Arable land and market gardens | I1 | 1441 |
| Moist or wet tall-herb and fern fringes and meadows | E5.4 | 734 | Waste deposits | J6 | 90 |

**Table S3** Comparison of coefficients of determination (R^2^) when using different phylogenetic resolution for the definition of the clade indices. Fit accuracy of species richness is included for reference. We examined the effect of phylogenetic resolution in the grassland dataset.

| Level | Richness | Divergence | Regularity |
| --- | --- | --- | --- |
| Species richness | 68.9 | 43.0 | 37.2 |
| Major clade | 74.8 | 88.2 | 65.0 |
| Order | 79.0 | 92.5 | 73.0 |
| Family | 80.7 | 95.8 | 81.4 |

**Table S4** Summary of linear models presented in Figure 2 (case studies). All terms in all models had significance < 0.001. For phylogenetic regularity, we fitted generalized least squares to acknowledge decreasing heteroscedasticity.

| Case study | Transformation | Terms | Equation | R^2^ (%) |
| --- | --- | --- | --- | --- |
| a) species-rich grasslands |  |  |  |  |
| Richness | sqrt | linear | -7.8 + 12.4x | 80.7 |
| Divergence | none | quadratic | -16.4 + 347.3x – 133.8x^2^ | 95.8 |
| Regularity | log | quadratic | 7.7 + 7.3x – 6.6x^2^ | 78.1 |
| b) CNPD |  |  |  |  |
| Richness | sqrt | quadratic | 9.9 + 1.4x + 0.07x^2^ | 79.2 |
| Divergence | none | linear | 10.5 + 244.9x | 94.4 |
| Regularity | log | quadratic | 9.4 + 3.5x – 4.3x^2^ | 54.9 |

**Table S5** R^2^ values (%) of the clade indices fitting all three dimensions of phylogenetic diversity separately in each herbaceous habitat in the CNPD. Habitat coding and number of plots in Table S2.

| Habitat | Richness | Divergence | Regularity |
| --- | --- | --- | --- |
| C1 | 70.8 | 90.5 | 18.3 |
| C2 | 72.6 | 96.8 | 46.5 |
| C3 | 64.2 | 96.7 | 42.8 |
| D1 | 85.6 | 95.3 | 43.0 |
| D2 | 78.1 | 90.9 | 58.1 |
| D4 | 73.4 | 94.3 | 87.2 |
| D6 | 65.2 | 92.7 | 79.1 |
| E1 | 73.6 | 92.6 | 82.8 |
| E2 | 65.8 | 93.5 | 77.3 |
| E3 | 66.8 | 93.8 | 74.3 |
| E4 | 62.2 | 74.4 | 77.3 |
| E5.2 | 68.8 | 93.8 | 83.6 |
| E5.4 | 71.1 | 92.9 | 54.7 |
| E5.5 | 65.5 | 91.2 | 35.0 |
| E5.6 | 70.6 | 93.2 | 49.1 |
| E6 | 85.7 | 92.3 | 52.1 |
| F2 | 71.2 | 88.8 | 47.4 |
| F3 | 74.6 | 87.6 | 53.4 |
| F4 | 87.9 | 90.9 | 45.7 |
| F9.1 | 86.1 | 95.5 | 69.7 |
| F9.2 | 78.4 | 84.7 | 24.3 |
| H2 | 51.9 | 90.9 | 55.2 |
| H3 | 61.3 | 94.7 | 72.9 |
| H5.6 | 80.2 | 93.0 | 80.7 |
| I1 | 74.2 | 92.2 | 77.3 |
| J6 | 57.8 | 94.8 | 25.7 |

**FIGURE LEGENDS APPENDIX**

**Figure S1** Phylogeny of 171 plant species occurring in 12 exclosures (long-term experiments) in species-rich grasslands. Posterior probabilities are indicated using different colors. Angiosperm super-orders and Ranunculales are labeled to allow orientation.

**Figure S2** Workflow of simulated community matrices used to assess fit accuracy of the proposed clade indices for all dimensions of phylogenetic diversity. We sampled species pools from three different clades (vascular plants, angiosperms, and super-asterids) from a megaphylogeny of vascular plants (Qian & Jin 2016). We randomly assigned species to species pools of various sizes (2000, 500, and 250 species). Finally, we created 50 community matrices for each specific species richness range, that is, six scenarios: 10–160, 10–80, 10–40, 10–20, 5–10, and 2–5 species per community. Species had randomly generated proportions. In total, we generated 2700 unique community matrices from 2700 unique species pools. For each matrix, we fitted a linear model with phylogeny-based metric (Faith’s PD, MPD, or VPD) as a response variable to its categorical alternative (clade indices, Table 2), and extracted a model R^2^. Each community matrix had 240 sites. Faith’s PD was always square-root transformed; VPD was always log-transformed.

**Figure S3** Phylogenetic richness (Faith’s PD) plotted against the clade richness index separately in each herbaceous habitat (Czech National Phytosociological Database dataset). Habitat coding in Table S2.

**Figure S4** Phylogenetic divergence (MPD) plotted against clade divergence index separately in each herbaceous habitat (Czech National Phytosociological Database dataset). Habitat coding in Table S2.

**Figure S5** Phylogenetic regularity (VPD) plotted against clade regularity index separately in each herbaceous habitat (Czech National Phytosociological Database dataset). Habitat coding in Table S2.

**Figure S6** Fit accuracy in simulated community matrices for a-c) phylogenetic divergence and d-f) phylogenetic regularity along the gradient of species richness ranges at different phylogenetic scales (super-asterids, angiosperms, and vascular plants).

**Figure S7** Dependence of phylogenetic diversity metrics and clade indices on species richness. a–b) Dependence of Faith’s PD and clade richness index (phylogenetic richness dimension) on species richness. c–d) Dependence of MPD and clade divergence index (phylogenetic divergence dimension). e–f) Dependence of VPD and clade regularity index on species richness (phylogenetic regularity dimension). Plots from both species-rich grasslands (black circles) and the Czech National Phytosociological Database (gray circles).

**REFERENCES APPENDIX**

Chytrý, M., & Rafajová, M. (2003). Czech National Phytosociological Database: basic statistics of the available vegetation-plot. Preslia 75, 1–15.

Davies, C. E., & Moess, D. (2003). EUNIS habitat classification. European Topic Centre on Nature Protection and Biodiversity, Paris. More information at https://eunis.eea.europa.eu/habitats.jsp
